# Using Narrative Evidence to Convey Health Information on Social Media: The Case of COVID-19

**DOI:** 10.2196/24948

**Published:** 2021-03-15

**Authors:** Anat Gesser-Edelsburg

**Affiliations:** 1 School of Public Health University of Haifa Haifa Israel; 2 Health and Risk Communication Research Center University of Haifa Haifa Israel

**Keywords:** health and risk communication, social media, narrative evidence, crisis, pandemic, misinformation, infodemic, infodemiology, COVID-19, policy, segmentation, barrier reduction, role models, empathy and support, strengthening self/community-efficacy, coping tools, preventing stigmatization, at-risk populations, communicating uncertainty, positive deviance, tailor messaging, targeted behavioral change

## Abstract

During disease outbreaks or pandemics, policy makers must convey information to the public for informative purposes (eg, morbidity or mortality rates). They must also motivate members of the public to cooperate with the guidelines, specifically by changing their usual behavior. Policy makers have traditionally adopted a didactic and formalistic stance by conveying dry, statistics-based health information to the public. They have not yet considered the alternative of providing health information in the form of narrative evidence, using stories that address both cognitive and emotional aspects. The aim of this viewpoint paper is to introduce policy makers to the advantages of using narrative evidence to provide health information during a disease outbreak or pandemic such as COVID-19. Throughout human history, authorities have tended to employ apocalyptic narratives during disease outbreaks or pandemics. This viewpoint paper proposes an alternative coping narrative that includes the following components: segmentation; barrier reduction; role models; empathy and support; strengthening self-efficacy, community efficacy, and coping tools; preventing stigmatization of at-risk populations; and communicating uncertainty. It also discusses five conditions for using narrative evidence to produce an effective communication campaign on social media: (1) identifying narratives that reveal the needs, personal experiences, and questions of different subgroups to tailor messaging to produce targeted behavioral change; (2) providing separate and distinct treatment of each information unit or theory that arises on social networks; (3) identifying positive deviants who found creative solutions for stress during the COVID-19 crisis not found by other members of the community; (4) creating different stories of coping; and (5) maintaining a dialogue with population subgroups (eg, skeptical and hesitant groups). The paper concludes by proposing criteria for evaluating the effectiveness of a narrative.

## Introduction

### Background

During disease outbreaks or pandemics, organizations must convey effective information that will cause members of the general public to cooperate with guidelines and even change their behavior, as in the need for social distancing and isolation during the COVID-19 crisis [1]. Moreover, policy makers must convey information to health care professionals, who must deal with new care conditions and social situations [2]. This information must go beyond factual information such as morbidity and mortality statistics. It must also provide explanations to help the public understand the rationale behind the guidelines as well as information to help population subgroups cope with social conditions such as loneliness and anxiety caused by lifestyle changes.

In communicating this information, health organizations must also address the psychological, sociological, economic, and political factors motivating the behavior of diverse population groups; therefore, conveying information, messages, and guidelines to the public becomes quite complex [3,4]. Moreover, in a media- and communication platform-saturated environment, if policy makers do not convey information that is relevant to people’s needs, the public will lose interest and turn to other resources and channels [5-7].

Health information can be conveyed as statistical evidence or as narrative evidence. Statistical evidence usually entails a dry summary of quantitative information about a sample of cases that can be generalized to an entire population [8]. This information is conveyed in a statistics-based and didactic manner that appeals primarily to cognitive considerations.

In contrast, health information can also be conveyed in the form of narrative, through stories that address both cognitive and emotional aspects. Narrative evidence is constructed in the form of a plot that has a beginning, a middle, and an ending that is often open [9-13]. Stories involve characters who portray incidents, life experiences, problems, conflicts, or questions, and challenges emerging from their daily lives or during crises. These characters transcend their personal stories to represent communal stories that often entail information about goals, plans, actions, and outcomes [9-13].

In examining whether narrative or nonnarrative [9,13-15] means are most effective in conveying health care information and creating health behavior change, research has uncovered apparently contradictory results. For example, one study found that narrative evidence is more effective than statistical evidence [16], whereas a meta-analysis indicated that statistical evidence is more persuasive [8].

In another meta-analysis, Zebregs et al [17] identified the influential factors in the two approaches: statistical evidence versus narrative evidence. Statistical evidence was found to exert a stronger influence on beliefs and attitudes than narrative evidence, whereas narrative evidence had a stronger influence on intention. The authors’ explanation was that statistical evidence, beliefs, and attitudes are mainly related to cognitive responses, whereas both narrative evidence and behavioral intention are more specifically related to affective (emotional) responses. Accordingly, during a pandemic or other crisis, policy makers can employ both means of information transmission: statistical evidence and narrative evidence. As noted by Zebregs et al [17], narratives can help influence people’s intentions to change their behavior, as required by unusual situations.

Hoper and Clippard [18] identified five qualities of narrative messages that make them particularly promising for health interventions. Narrative messages can overcome resistance toward the advocated health behavior. Moreover, they can engage audiences that are less involved, reach audiences that are less knowledgeable, render complex information comprehensible, and ground messages in the culture and experiences of the target audience. In the next section, we describe these five qualities and tie them to the field of emerging infectious disease communication. We also add two qualities that we believe are of particular importance for the use of narrative strategy in health communication. The first is using aesthetic means (nonverbal communication) to convey information based on the edutainment theoretical framework [19,20], as such means are important components of persuasion strategies. The second is conveying a diffused story through social networks based on the diffusion of innovations theory [21,22] and parasocial interactions [23-25], both adapted to the current social media realm.

### Overcoming Resistance to Advocated Health Behavior

Resistance can be broadly defined as a reaction against change or an incentive to oppose persuasive appeals. Resistance to persuasive messages may include counterarguing the message’s claims, ignoring the message altogether, or denying the validity of the message due to its source. The greater the public’s resistance, the greater the advantages of the narrative approach in reaching people [26].

### Rendering Complex Information Comprehensible

Narrative evidence helps people process new or complex information by putting the facts into the context of a specific time and place during an outbreak or pandemic. Moreover, narratives can be used to link the information to the experience of the readers or listeners [27,28].

### Reaching Audiences That Are Less Knowledgeable

Policy makers seeking to find the most effective way to lead the public to heed information and guidelines during a crisis need a tool that does not require a high level of literacy or education. Narrative evidence meets that requirement because it can address people at all levels and in all languages [18].

### Engaging Audiences That Are Less Involved

Addressing emotions is one way to make the public feel involved. Emotions have long been acknowledged as an essential ingredient in the recipe for persuasion [29-31]. In the health communication field, persuasive messages that arouse emotions tend to be perceived as more effective than those that do not [32-36]. Even health care workers may be more responsive to messages that address their emotions than to statistical data. Hence, this approach can be used to provide health care workers with tools and vital information to help them communicate with families whose loved ones are hospitalized with COVID-19.

### Grounding Messages in the Culture and Experiences of the Target Audience

People exposed to other people’s stories in the media undergo a “parasocial relationship” [37] in that they become engaged with the characters despite never having met them. In line with social-cognitive theory [38], such characters may serve as role models for appropriate behavior by demonstrating the costs and benefits of different courses of action. People may be inspired to imitate the actions of positive characters, avoid the problems of negative characters, or follow in the footsteps of characters who undergo a transformation (usually from negative to positive attitudes or behaviors) over the course of the story [39].

In media campaigns, the characters in a narrative can serve as role models for the readers or viewers [40]. For instance, characters representing at-risk population groups can depict different coping situations that the public can learn and imitate [41]. As opposed to merely short text (eg, a Tweet that can contain up to 280 characters), social media platforms such as Facebook, blogs, or COVID-19 forums enable people to create and share their stories. These platforms provide people with opportunities to talk about their fears and concerns as well as their beliefs and risk perceptions in different situations [5,42,43]. Policy makers can study stories on social networks to learn how people understand the epidemic narrative at any given time and use that information to generate appropriate narratives.

Stories can be used for long-term interventions as well as for the short-term needs of a specific context, such as the outbreak of a pandemic. Furthermore, pandemics are not necessarily short. Indeed, the COVID-19 crisis is prolonged and ongoing. For example, policy makers can design stories that model effective behavior for the subgroup of health care workers caring for older people during the COVID-19 crisis.

### Using Aesthetic Means to Convey Information

The newly proposed quality of aesthetic means is of particular importance in health communication. Narratives can use aesthetic means and strategies such as empathy, humor, sarcasm, and irony to convey information using [27]. Edutainment has shown that aesthetic experiences provide viewers with opportunities for meaningful cognitive illumination or change in the context of health or other issues [19,20]. Aesthetic means offer added value that cannot be achieved merely by conveying statistical information.

### Conveying a Diffused Story Through Social Networks

Conveying a diffused story [21,22] through social media leads to parasocial interactions [23-25] and generates relationships between people that transcend geographic and linguistic borders. These relationships turn strangers into friends and transform passive audiences into active coparticipants [44,45]. People who hear a good story can be expected to share it with others, initiating a pattern of social proliferation, such that messages “go viral” [46,47]. Thus, the boundaries between the personal and the public become blurred. For example, when people identify with a story posted on social networks and share it on their feeds, they actually turn that story into “their” story. Hence, one individual’s story becomes the story of many other individuals, who identify with it and share it with others.

This viewpoint paper combines three intertwining parts to provide a holistic perspective on the use of narrative evidence during a disease outbreak or pandemic. The first part compares the commonly used strategy of apocalyptic narratives to the more desirable strategy of coping narratives, an alternative that has not yet been fully implemented. The second part outlines the conditions necessary to generate an alternative coping narrative and discusses the outcomes of this alternative. Finally, the third part proposes evaluation criteria that can be used in constructing an alternative coping narrative.

## Objectives

### First Objective

The first objective of this viewpoint is to introduce policy makers to the advantages of using narrative evidence [9,13-15] during a disease outbreak or pandemic, such as the COVID-19 pandemic. To date, health organizations have used narratives mainly in the fields of clinical care and education. These narratives usually focus on disease prevention, disease management, patient recovery, and psychological and social resilience [17]. However, using narrative evidence as a tool for changing attitudes and behaviors can be effective not only for long periods of clinical care intervention but also for shorter periods that require the public to change its behavior.

### Second Objective

The second objective of this viewpoint is to propose an alternative coping narrative based on health and risk communication approaches and models. Throughout human history, authorities have tended to employ apocalyptic narratives that include threats, intimidation [48], and the use of “good” and “bad” protagonists. However, alternatives are available to this apocalyptic narrative.

### Third Objective

The third objective of this viewpoint is to propose five conditions for constructing and using alternative narrative evidence to launch a communication campaign on social media.

### Fourth Objective

The fourth objective of this viewpoint is to propose criteria for evaluating a narrative’s effectiveness and potential to generate change: narrative mechanisms, rhetorical concerns, and empirical questions.

Based on the aforesaid, policy makers can use narrative evidence not only for long-term interventions but also during disease outbreaks and pandemics when members of the public are called upon to follow guidelines and change their behavior. In the next section, we propose an alternative coping narrative model instead of the apocalyptic narrative model commonly used during disease outbreaks and pandemics.

## Traditional Use of Narrative Evidence in Pandemics: Apocalyptic Narratives

Pandemics are difficult and complex events with a high level of uncertainty. From the dawn of history, pandemics have aroused fear, panic, and alarm, as expressed in many Western works of literature and art [49-54]. Over the years, human and technological progress has led to the development of vaccinations. Nevertheless, epidemics and pandemics, such as the COVID-19 pandemic, still pose a serious challenge, with wide-ranging existential consequences that spark primeval emotions and fears. Questions arise [55-58] such as “How can leaders deal with the public’s fears, uncertainty and concerns?” and “What narrative can policy makers create in the public sphere to gain people’s trust and cooperation?”

Some, though not all, health organizations currently employ apocalyptic narratives [59]. This sort of narrative lacks many of the qualities of narrative evidence while also containing some elements that can generate negative responses among the public. In this section, we describe the features of apocalyptic narratives traditionally used during pandemics and discuss why these have not been effective. After that, we describe an alternative coping narrative based on the health and risk communication literature that some countries have put into effect during the COVID-19 pandemic.

Throughout human history, pandemic narratives have incorporated melodramatic and apocalyptic features [59,60]. Indeed, the word “epidemic” refers to something that “falls upon people“ (in Greek, *epi* means ”upon or above“ and *demos* means ”people“) [61]. Hence, by definition, epidemics are unpredictable and are therefore perceived as threatening.

Artistic expressions of epidemics in literature, painting, sculpture, and other media symbolize the sense of vulnerability in the face of uncertainty and death, as well as the random nature of death itself. The villain of the plot is the virus that is threatening to destroy humanity, while the “good guys” or heroes are the lifesaving medical workers. The narrative also includes characters depicted as disease spreaders, usually from disempowered communities. Blame, stigmatization [62], and fears and anxieties (whether real or exaggerated) swirl around in the public consciousness [62]. The tone of this narrative is apocalyptic rather than redemptive. Diseases are managed and endured rather than overcome, and species-level damage is incurred. In pandemic narratives, our anxieties are not assuaged; we are invited to struggle rather than to overcome.

According to Wald, pandemic narratives tell “a contradictory but compelling story of the perils of human interdependence and the triumph of human connection and cooperation, scientific authority, and the evolutionary advances of the microbe, ecological balance, and impending disaster” [62]. Further, Massumi [63] indicated that we live in an environment that is not so much threatening as “threat generating” [64]. That is, the threat is not always as existential as its effect on human consciousness, as expressed through the stories we tell.

In the modern pandemic narrative, traditional and social media do not only cover and mediate the crisis; they also serve as narrators that dictate the reality and narrative of the pandemic to the audience. In this narrative, humanity searches for a solution in the form of a medication or vaccination that will redeem it from the apocalyptic threats [3]. In recent disease outbreaks, health organizations seem to have strengthened this apocalyptic narrative by using strategies of intimidation to make the public follow instructions and guidelines [3]. This can be seen in the language and tone of information delivery (eg, use of war language to describe COVID-19 as a cruel enemy that needs to be defeated [65]).

Moreover, the modern pandemic narrative often uses overblown statistics not backed by accurate facts to describe morbidity and mortality to motivate the public to follow directives. For example, Dew [66] describes how during the 1997 measles outbreak in New Zealand, the Ministry of Health ran a media advertisement campaign using emotional appeals and employing statistics and numbers to create a “quantification rhetoric.” According to Petersen and Lupton [67], this rhetoric “tends to suggest the figures used are not subject to doubt or uncertainty.” During the media campaign, ”the viewer was subjected to images of cemeteries and crucifixes passing across the screen” [66]. However, the 1997 outbreak in New Zealand was found to be minor. The actual number of measles cases reported was 1200, and not a single child died [66]. Intimidation has also been used during the COVID-19 pandemic. For example, the prime minister of Israel compared the first wave of the epidemic to both Spanish influenza and the Holocaust, citing inaccurate statistics [68,69].

Policy makers and organizations often tend to frame uncertain information in terms of certainty. Their assumption is that uncertain information may create negative emotions. Furthermore, even when the risk is uncovered, often through social media, and its communication becomes inevitable, experts and organizations are often reluctant to reveal all available information. They prefer to provide a straightforward and unambiguous explanation. Van Asselt et al [70,71] called this framing “the uncertainty paradox,” referring to situations wherein uncertainty is acknowledged, but the role of science is framed as providing certainty [72].

Contrary to this assumption, other studies indicate that when people feel they do not have sufficient information regarding a risk, their sense of uncertainty and negative feelings may increase [73-76], especially when the risk is perceived as severe and uncontrollable [77]. Indeed, honest risk communication and providing sufficient information do not have a negative impact on the public’s behavior. In contrast, sufficient and accurate information can help mitigate negative feelings [78-81].

Authorities often use intimidation strategy because they believe the public is in a state of “panic” and “hysteria” during a crisis [82]. For example, the public’s reaction to the appearance of four Ebola cases in the United States and to the authorities’ diverse approaches to necessary precautionary measures was perceived as “national panic” [83,84], with Maryn McKenna [85] coining the term “Ebolanoia” to describe it.

Contrary to this widely accepted view of public panic, empirical studies of public response to extreme situations have revealed the opposite findings [79,80,86]. Indeed, some studies indicate that in extreme situations, people are more likely to react by demonstrating social cohesion and mutual trust rather than showing panic [87].

Even in the case of public panic, using intimidation without empowering individual self-efficacy is counter to the theory of intimidation use known as the extended parallel process model (EPPM) [88]. The EPPM attempts to predict how individuals will react when confronted with fear-inducing stimuli. For fear-based policies to be effective, policy makers must induce a moderate level of fear alongside a higher level of self-efficacy and response efficacy. When the public’s fear exceeds its sense of self-efficacy, the message becomes ineffective.

## An Alternative Coping Narrative

As opposed to this apocalyptic narrative, here, we propose an alternative coping narrative based on health and risk communication approaches and frameworks [89,90]. This narrative should contain the following components: segmentation, barrier reduction, role modeling, empathy and support, tools to promote self and collective efficacy and coping, preventing the stigmatization of at-risk populations, and communication of uncertainty.

### Segmentation Through Narrative

The literature underscores the importance of segmenting [91,92] and mapping [93,94] each subgroup in the population to tailor [95,96] the information and media campaign to the barriers, risks, concerns, and unique needs of each group. During every disease outbreak or pandemic, some groups are at higher risk than others. The narrative put forward by the authorities must communicate and distinguish between actions taken for the benefit of the public at large and those targeting specific at-risk groups. For example, during the COVID-19 pandemic, young people between the ages of 18 and 30 years without any underlying conditions are at lower risk of serious illness. Therefore, the authorities must tailor risk messages to particular at-risk groups without resorting to intimidation.

### Reducing Barriers Through Narrative

The strategy of barrier reduction entails reducing existing difficulties and barriers to the adoption of desirable behavior [97,98] and offering incentives and solutions to the population. This strategy can be useful during a disease outbreak or pandemic. One of the barriers to adoption of desirable behavior during the COVID-19 pandemic is the difficulty of maintaining social distancing. By means of narratives that illustrate this barrier while providing ways of coping with it, the public can be given solutions for complying with social distancing without the use of intimidation.

### Role Modeling Through Narrative

Research has shown that role models, identification, and social support can be used effectively in interventions to change health behavior [38,99]. According to Bandura’s sociocognitive theory, individuals can learn a behavior by observing a model. Moreover, they will be more likely to perform this behavior if they see positive and appealing reinforcement for the behavior. The use of role models boosts self-efficacy in that the characters demonstrating a particular health behavior provide viewers with tools and skills.

The use of role models to teach social behavior through narratives can be implemented during disease outbreaks or pandemics as well. During the COVID-19 pandemic, for instance, narratives using positive role models can demonstrate the advantages of following the guidelines, thus strengthening people’s self-efficacy. Likewise, patients who survived COVID-19 can share their experiences and give tips to the public. Leaders dealing with the crisis can also serve as role models through their behavior. For example, during the COVID-19 crisis, New Zealand’s Prime Minister Jacinda Ardern announced a 20% salary cut for herself and the members of her cabinet [100].

### Strengthening Collective Efficacy Through Narrative

Beyond strengthening individual self-efficacy, narratives can strengthen collective efficacy by illustrating the community’s ability to provide social support for its members. A community’s collective efficacy can be reinforced through stories that emphasize solidarity and mutual support for weaker community members during a health crisis or pandemic. For example, during the COVID-19 pandemic, civic organizations and individuals can support older people under lockdown by helping them obtain food and medicine.

### Using Narratives to Prevent Stigmatization of At-Risk Populations

During a health crisis, authorities sometimes worry that at-risk population groups will reject relevant information for fear of being stigmatized by the media and society. The literature points to the possibility of self-stigmatization or social stigmatization if media outlets use sensational means to communicate a risk [101]. For example, during the COVID-19 pandemic, the Asian American community expressed strong fears of being blamed for the spread of SARS-CoV-2. Therefore, policy makers should stress stigma reduction and create narratives that underscore social solidarity.

### Communicating Uncertainty Through Narrative

Scholars investigating the topic of risk found that dealing with uncertainty is a major challenge in risk assessment and management. According to Frewer et al [102], public health experts tend to believe that the public is incapable of coping with the uncertainties associated with risk management. Contrary to this opinion, studies in the risk communication literature indicate that in risky situations [70,71,103,104], especially those that involve uncertainty [105], the public wants full information transparency. Transparent communication does not provoke negative reactions among the public; rather, it helps reduce negative feelings and increases the public's respect for the risk-assessing agency [79].

Sandman and Lanard [106] emphasize the need to “proclaim uncertainty,” advising authorities to disseminate tentative information if it is the only type of information they have. A number of studies conducted during pandemics, such as the Ebola outbreak in the United States [107] and the polio outbreak in Israel [108], reinforced Sandman and Lenard’s hypothesis by showing that the public wants organizations to communicate uncertainty. Furthermore, the public becomes impatient and uncooperative when authorities only give them partial or selective information [109].

**Figure 1:**
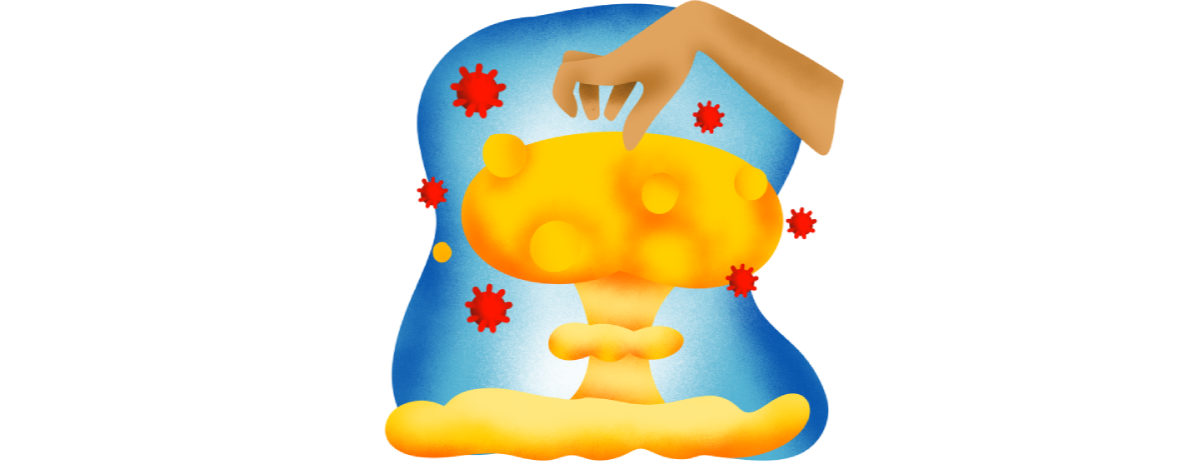
An apocalyptic pandemic narrative.

**Figure 2:**
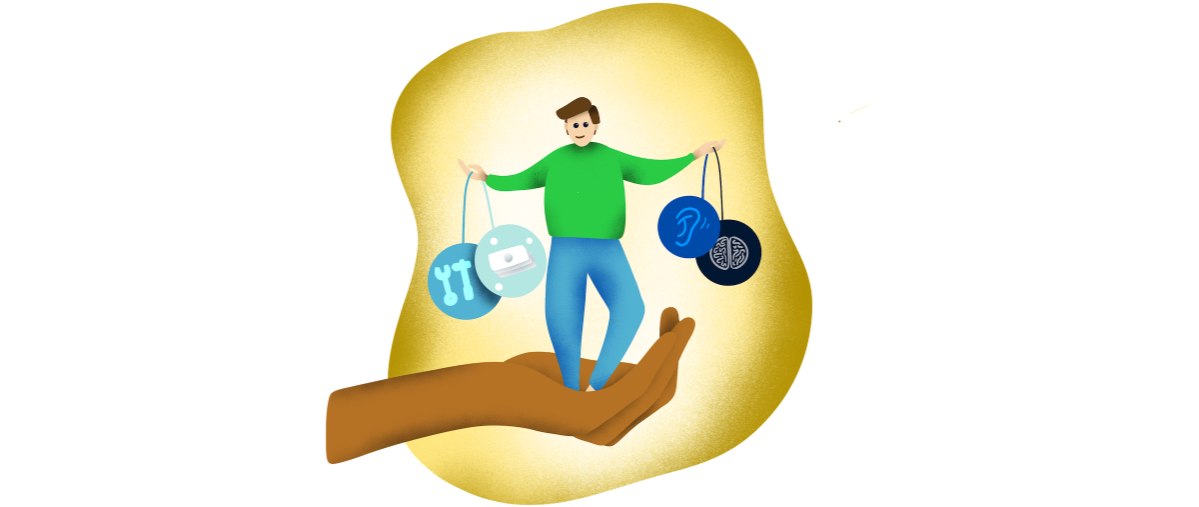
An alternative pandemic narrative that presents coping strategies.

Table 1 summarizes the strategies and components of a pandemic coping narrative based on health and risk communication approaches. For each apocalyptic pandemic narrative, an alternative pandemic narrative that offers coping strategies is presented to help health organizations transform one narrative to the other. Figure 1 depicts an apocalyptic pandemic narrative, in which COVID-19 is depicted as an apocalyptic explosion of an atomic bomb causing severe harm to humans. Figure 2 depicts an alternative pandemic narrative that offers coping strategies, using additional tools provided to people to cope with the COVID-19 pandemic.

In this section, we explained how an apocalyptic narrative can be transformed into a coping narrative. In the next section, we propose several conditions necessary for constructing and using a coping narrative to launch a communication campaign on social media.

**Table 1:** Apocalyptic pandemic narratives versus alternative coping narratives.

Apocalyptic pandemic narrative	Alternative pandemic narrative that offers coping strategies
Waging war against an enemy	Coping with situations of uncertainty
Using intimidation strategies to motivate the public to follow guidelines	Using empathy strategies and reflexivity to motivate the public to cooperate
Creating heroes (leaders/life-saving medical teams)	Creating social support and mutual aid through health organizations
Prioritizing public health as the most important thing	Differentiating between public health and personal risk
Taking extreme measures to protect the public	Introducing fact-based measures
Using sensationalism and dramatization	Seeking truth and exposing policy makers’ doubts and questions
Enacting surveillance, guidelines, and regulations (“Big Brother”)	Transparency and rationalization of guidelines
Stigmatizing and blaming groups that do not follow guidelines	Encouraging solidarity
Closed ending: defeat or victory over the virus	Coping and living in a changing and dynamic situation

## Use of Narrative Evidence to Communicate About COVID-19 via Social Media While Maintaining Constructive Dialogue With the Public

A narrative media campaign launched on social media can be based on one or both of the following methods: (1) posting personal stories on social networks and distributing them to relevant subgroups in the population via channels targeting these groups; (2) using narratives based on preliminary research that identifies the public’s questions and concerns and responds to them through narrative evidence posted on social networks. Each of these methods requires five main conditions. In the following section, we outline these conditions, methods, and prospective outcomes. These conditions and their outcomes are formulated based on integrating health and risk communication theories [3,4,110]. We thus provide a new perspective on the use of narrative and communication strategies during disease outbreaks and pandemics—in this case, the COVID-19 pandemic. These conditions and outcomes have a high degree of reliability and can be further validated by additional empirical research.

### First Condition: Tailor Messages Toward Targeted Behavioral Change Based on the Needs and Experiences of Different Subgroups

Despite the theoretical understanding that national health authorities should build segmented profiles of their publics [111], this understanding has not yet been fully implemented. During the midst of the H1N1 pandemic, countries were called on to adapt their communication strategies to specific cultural needs [112], pointing to a general lack of such cultural and social adaptation [111]. Although government agencies have long recognized the ineffectiveness of one-size-fits-all messaging [113], studies have indicated that segmentation is still far from adequate implementation [114-118].

#### Method

Content analysis [119] and ethnographic analysis [120] should be used to map and categorize the narratives of specific subgroups on social media. The variables defining such groups will vary depending on the issue. In addition to sociodemographic or geodemographic variables typifying different countries, other variables will be based on specific attributes of different groups (eg, trust in authorities, science skepticism, and vaccination hesitancy). Through this research apparatus, policy makers can use qualitative and quantitative tools to map and analyze the stories arising from different population groups and the theories to be elicited from them.

#### Outcomes

**Figure 3:**
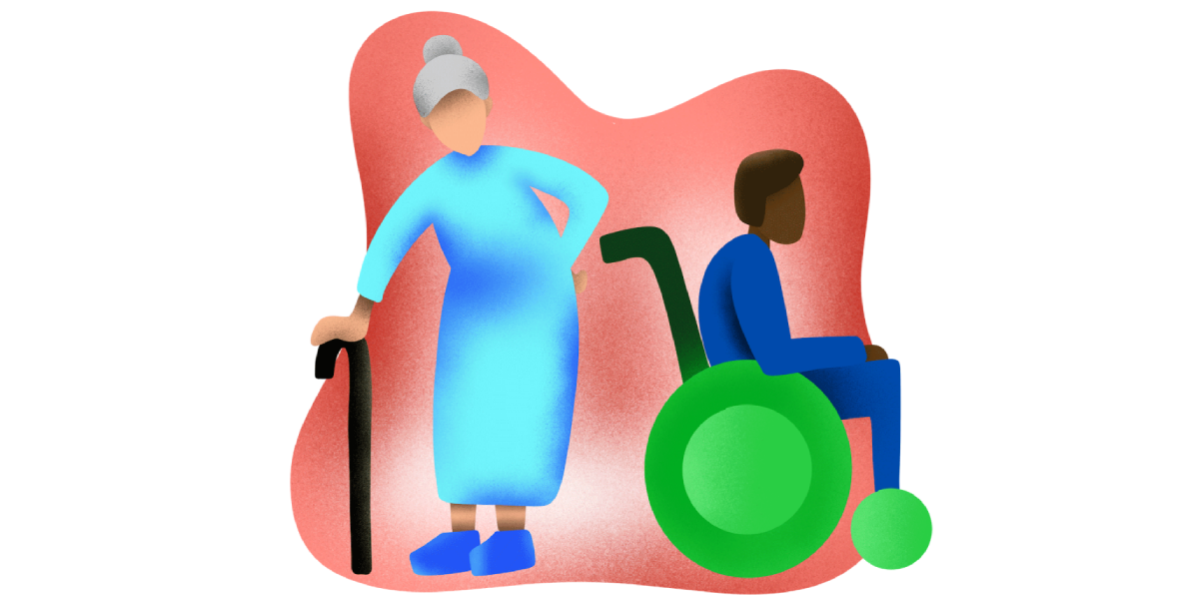
Identifying the main stories of each subgroup.

Health organizations will be able to understand the needs, needs, assumptions, and risk perceptions of different groups and respond instantly. When health organizations identify the main stories of each subgroup, they will be able to adjust the relevant information accordingly. Figure 3 depicts the need to identify the main stories of each subgroup, such as older or disabled people.

Health organizations can use people’s authentic stories to disseminate essential information to the community. When health organizations use the experiences of people who found ways to cope with different crisis situations, other people can learn from that information, thus building social resilience.

### Second Condition: Refer Separately and Distinctly to Each Information Unit or Theory Arising on Social Networks

Studies show that when health organizations want to communicate facts to the public, they often distinguish between myth and fact [121-123]. This distinction is not neutral and has been found to be ineffective for two reasons. First, when information provided on a website is identified as a myth, people still remember the information, even though it is totally or partially untrue. Second, the public refuses to accept a judgmental approach without scientific evidence. In two studies on public attitudes toward the measles-mumps-rubella vaccine and the seasonal influenza vaccine [124,125], pro-vaccine information from the US Centers for Disease Control website had a “backfire effect.” After being given information intended to refute the supposed connection between vaccinations and autism, vaccine skeptics formed even stronger negative opinions about vaccinations.

Health organizations must provide separate and distinct treatment for any kind of information unit or theory that arises on social networks. For example, social media platforms are filled with rumors pertaining to COVID-19 [126,127]. Health organizations have generally used a single approach to handle information they consider unfounded, without sufficient differentiation. Thus, they countered the claim that the virus originated in a laboratory in Wuhan in the same way they countered the proposal to eat garlic as a cure or the notion that the virus can be killed through exposure to sunlight. These units of information differ, and each deserves to be engaged and addressed on its own merits.

#### Method

Answer the questions and theories posed by different population groups, not by correcting the information but rather by differentiating the information and addressing each claim on its own merits.

#### Outcome

**Figure 4:**
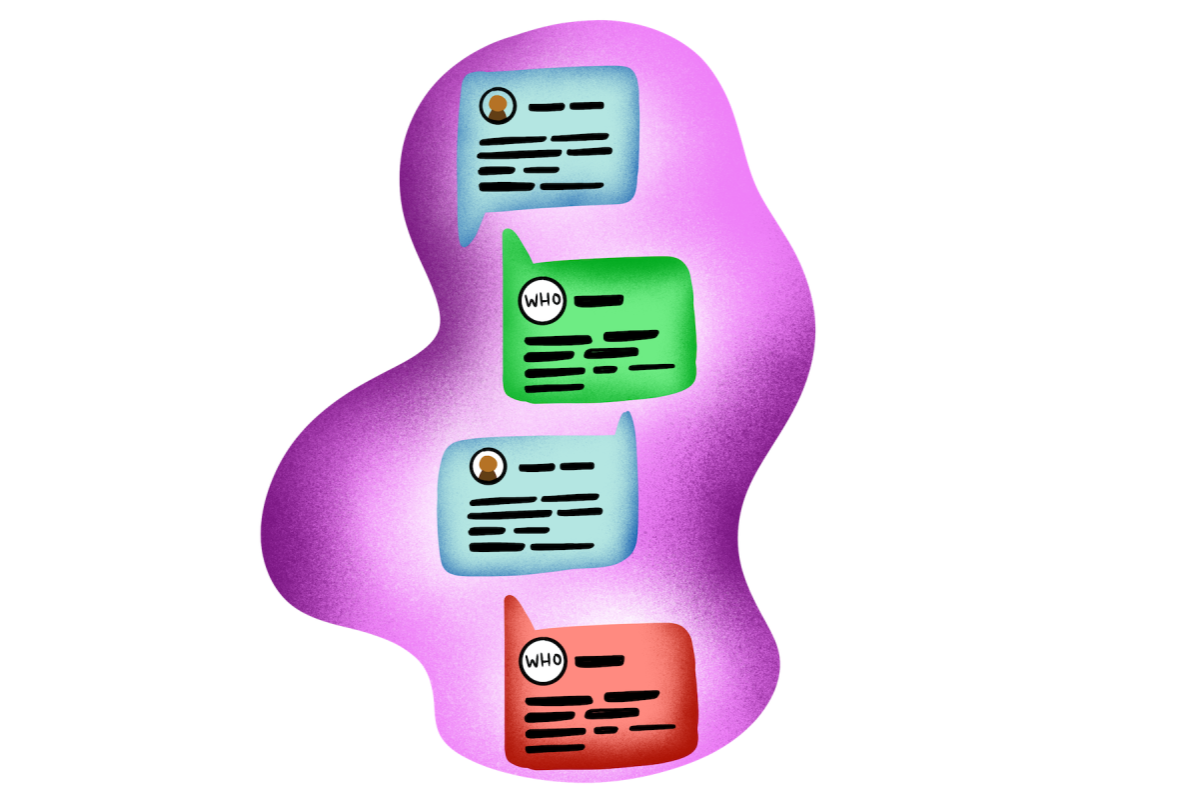
Answering the questions and theories posed by different population groups.

By distinguishing among different theories that arise on social media and addressing them separately, health authorities will build the public’s trust. Health and risk communication theories show that bidirectional dialogue is critical, that is, the positions and arguments emerging from a theory should be addressed through a dialogue between equals. Likewise, conveying positive feedback regarding the factual parts of different theories raised on social networks will give the public a sense of transparency and trust. In contrast, deciding to correct or dismiss entire theories (including their correct parts) can generate antagonism, such that the public feels its views are being dismissed. Figure 4 depicts the need to conduct a dialogue between health organizations and the public regarding COVID-19–related concerns and questions raised by the public.

### Third Condition: Identify Positive Deviants That Offer Creative Solutions

According to Singhal [128], “the Positive Deviance (PD) approach is based on the premise that in every community there are certain individuals or groups whose uncommon behaviors and strategies enable them to find better solutions to problems than their peers, while facing worse challenges and having access to the same resources. However, these people are ordinarily invisible to others in the community.” The PD approach seeks to identify and streamline existing resources deriving from within a community rather than to import external “best practices.” Such practices are distributed and implemented over time via social networks [129,130].

Health organizations should seek out positive deviants [131-133] who propose creative (“outside the box”) solutions for stressful situations emerging from the COVID-19 crisis that other members of the community did not find.

#### Method

Health organizations should use the narratives of exceptional individuals in various groups who have found ways to cope with loneliness, stress, and pressure. These coping means can then be disseminated to other members of their community.

#### Outcome

**Figure 5:**
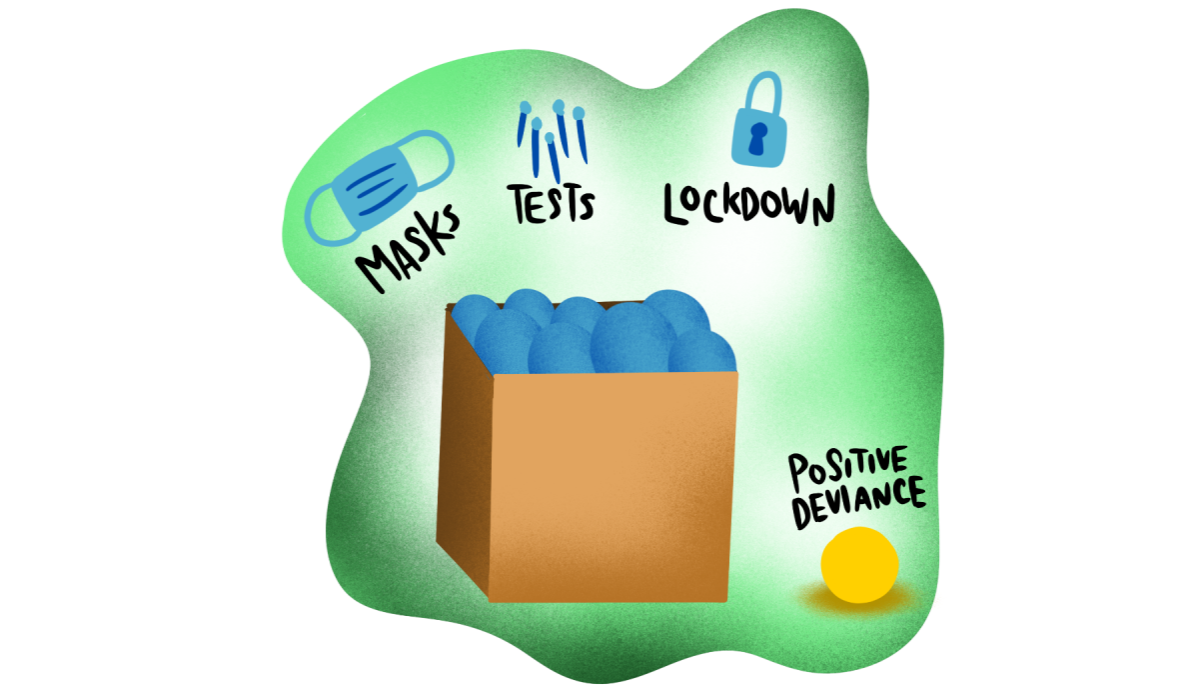
Thinking outside the box.

These creative solutions and “thinking outside the box” will generate role models and promote tips from ordinary people representing various population groups that can help the public cope during the COVID-19 crisis. The advantage is that the community is more likely to accept solutions coming from inside than those imposed by the authorities. Figure 5 visualizes the need to think outside the box to find creative solutions that can be adapted to the changing state of the COVID-19 pandemic.

### Fourth Condition: Create Different Stories of Coping Experiences

Storytelling relies upon realism, identification, and transportation to help people understand different points of view and change their attitudes and health behavior [134]. According to Lee et al [134], “narrative communication is context-dependent because it derives meaning from the surrounding situation and provides situation-based stories that are a pathway to processing story content.”

#### Method

**Figure 6:**
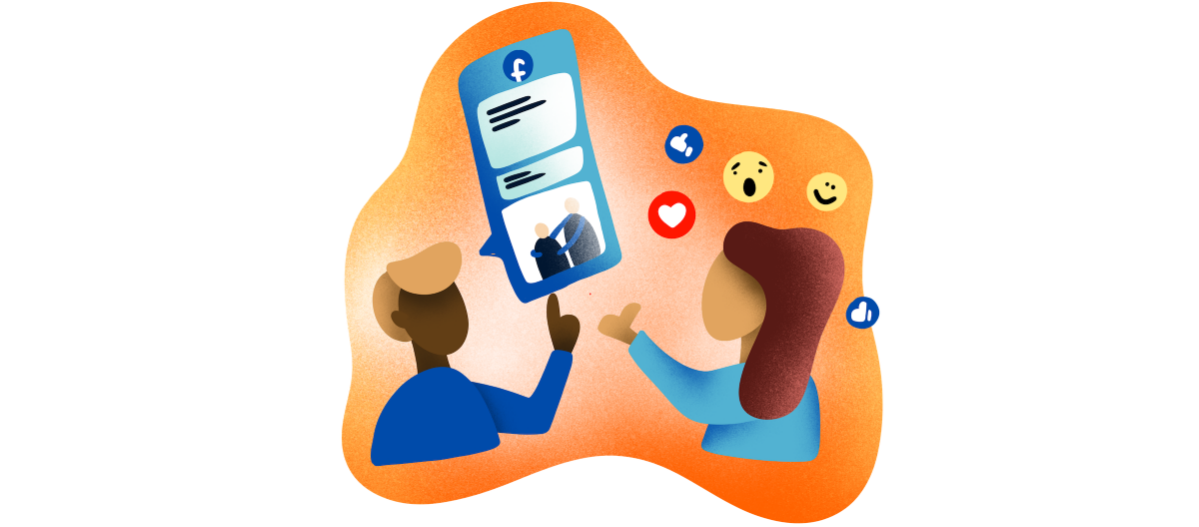
Creating different stories of coping experiences.

Instead of the dry statistics and didactic guidelines that health authorities convey, members of different subgroups can share their stories with their friends and introduce dilemmas and emotions emerging from their coping experiences. Figure 6 visualizes the need to create different coping stories using strategies such as identification and humor.

These narratives may be in the form of testimonials. They can also be dramatizations of personal narratives [13] that illustrate what happened to the narrator or to other individuals during the crisis (eg, a story about how a patient from an immigrant group copes with stress).

#### Outcome

These stories can provide specific tools to help different population subgroups cope with the crisis. 

### Fifth Condition: Maintain a Dialogue With Skeptical and Hesitant Groups

According to Larson [135], “educational materials and resources are important, but limited; health officials and educational campaigns often fall short because they craft messages based on what they want to promote, without addressing existing perceptions. Dialogue matters. Strategies must include listening and engagement. We have to get better at this…”

#### Method

Health authorities can use the authentic narratives and social media posts of skeptical and hesitant groups to answer questions and address arguments while providing objective and transparent information. In doing so, authorities should not attempt to frame the arguments of these hesitant and skeptical groups in terms of myths versus facts or as misinformation.

#### Outcomes

**Figure 7:**
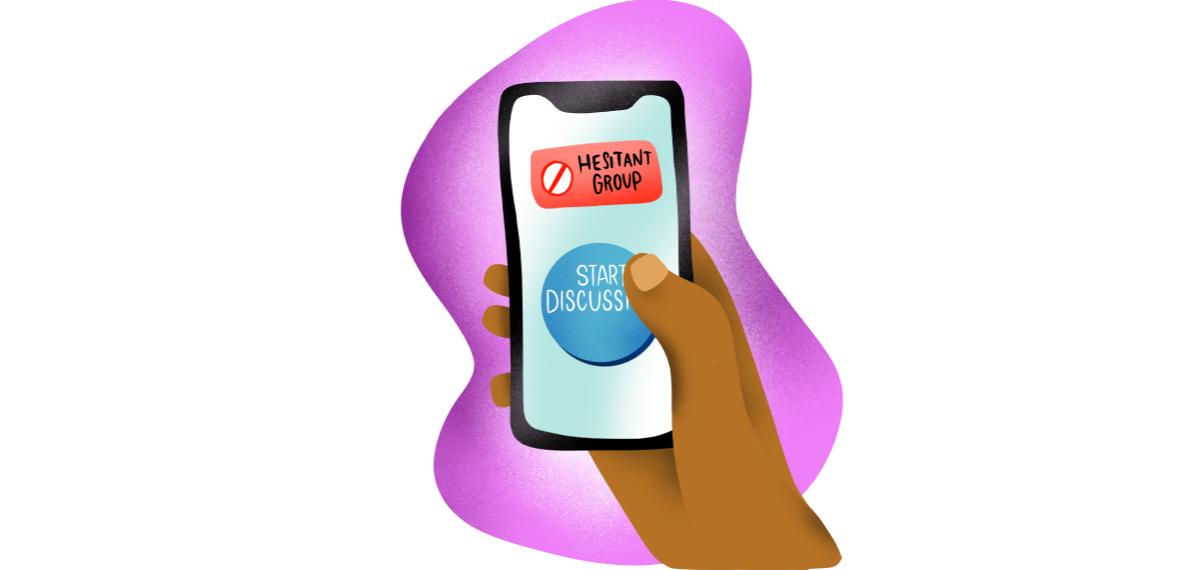
Maintaining a dialogue with skeptical and hesitant groups.

Building trust among skeptical groups will have consequences for enlisting the cooperation of these groups in future pandemics. Figure 7 depicts the need to maintain a dialogue with skeptical and hesitant groups.

After outlining the conditions underlying the use of narrative evidence to communicate crises, we now propose criteria for evaluating the effectiveness of a narrative.

## A Formative Evaluation Toolkit for Health Organizations

Formative evaluation of a narrative must take into consideration both the narrative created by the organization itself and the authentic narratives found on social networks and used by the organization during campaigns. The purpose of formative evaluation is to ensure that the intervention element is applicable, suitable, significant, and acceptable to the program’s target audience [136]. Formative evaluation focuses on participatory research with the target audience before, during, and after launching the communication campaign. It includes checking the barriers, needs, and preferences of the target audiences and setting objectives on the way to designing the narrative. Formative evaluation for narrative building should be preceded by qualitative empirical research among representatives of the target audience (including personal interviews, focus groups, and role playing).

Table 2 summarizes the questions and issues relevant to examining a narrative through evaluation research.

**Table 2:** A rhetorical matrix for empirical analysis of narrative mechanisms and potential for change (evaluation toolkit), based on Gesser-Edelsburg and Singhal (2013) [19].

Narrative mechanism	Rhetorical concerns	Empirical questions to gauge a narrative’s potential for change
Dialogue (between the narrative and the public)	How do the produced messages and dialogue engage with the public’s predisposed realities?	In processing the narrative, to what extent did the public feel They were invited or coerced into a dialogue about coping with the challenges? The messages were consensual or oppositional to their predispositions? New possibilities for coping were raised in the narrative?
Involvement (the public’s emotional engagement with the narrative)	How is the public emotionally involved, immersed, or absorbed in the unfolding narrative?	In processing the narrative, to what extent did the public experience Feelings of voyeurism, empathic identification, alienation, or anger? Identification with certain characters, and how did that influence their perceptions and positions on the issues the characters represented?
Trust (public’s perceptions of the narrative’s credibility)	How does the public perceive the plausibility, realism, and veracity of the unfolding narrative? Is the narrative trustworthy? Credible?	In processing the narrative, to what extent did the public feel the narrative was credible? Realistic? Plausible? At what stage did the public begin to experience clarification of doubts and new emergent possibilities? What conditions facilitated this change?
Catharsis and transformation (narrative’s influence on the public)	How does public engagement with the narrative lead to new learning, alternative positions, and change possibilities? How does the modeling and reinforcement of change through characters increase audience motivation and self-efficacy for practice?	In processing the narrative, to what extent did the public feel They identified with the transformation of characters in the unfolding story? They went through a process of change parallel to the transformed characters? They were engaged and empowered by the characters and their story? The alternatives presented in the narratives are applicable to the reality of their behavior?

## Conclusions

The use of narrative evidence as a tool for changing attitudes and behaviors is effective not only for long periods of clinical care intervention but also for short ones, because in either case, the public is required to change its behavior. As we have realized during the COVID-19 pandemic, the public will be forced to change its lifestyle over the long term.

During a disease outbreak or pandemic, policy makers must deal with the flow of information on multiple media forums. Indeed, policy makers must compete for the public’s attention with other sources that may be manufacturing misinformation. In such a complex multimedia environment, the use of narrative has many advantages.

Seven qualities of narrative messages make them particularly promising for health interventions. Narrative messages can overcome resistance toward the advocated health behavior, engage audiences that are less involved, reach audiences with less knowledge, render complex information comprehensible, ground messages in the target audience’s culture and experience [18], use aesthetic means, and convey a diffused story over social networks.

Throughout human history, authorities have tended to employ apocalyptic narratives during disease outbreaks or pandemics. This viewpoint paper proposes an alternative coping narrative model based on health and risk communication approaches and models incorporating the following components: segmentation [137]; barrier reduction [97,98]; role models, empathy, and support [90,99]; strengthening self-efficacy, community efficacy, and coping tools [89]; preventing the stigmatization of at-risk populations; and communicating uncertainty.

In this viewpoint paper, we also recommend five conditions for using narrative evidence that will lead to launching an effective communication campaign on social media:

1. Identifying narratives that reveal the needs, personal experiences, and questions of different groups to tailor messaging toward producing targeted behavioral change
2. Offering separate and distinct treatment of each information unit or theory of any kind that arises on social networks
3. Identifying positive deviants [131-133] who have found creative solutions for stress during the COVID-19 crisis that other members of the community did not find
4. Creating different stories of coping experiences
5. Maintaining a dialogue with subgroups (eg, skeptical and hesitant groups)

Evaluating the narrative constructed by health organizations is also very important. In this viewpoint paper, we offer criteria for evaluating the effectiveness of a narrative by addressing narrative mechanisms**,** rhetorical concerns, and empirical questions to gauge each narrative’s potential for change.

The proposed use of narrative as a communication tool will help policy makers more effectively manage how they communicate with the public during disease outbreaks and pandemics. Narrative is a human and pluralistic means that appeals to everyone. Hence, by using existing narratives on social networks while simultaneously creating new narratives to transmit information, health officials and policy makers are more likely to be able to influence actual health attitudes and behaviors.

## Abbreviations

EPPM: extended parallel process model
PD: positive deviance
